# Management of difficult airway in intratracheal tumor surgery

**DOI:** 10.1186/1472-6815-5-4

**Published:** 2005-06-07

**Authors:** Amit Goyal, Isha Tyagi, Prabhat Tewari, Surendra K Agarwal, Rajan Syal

**Affiliations:** 1 Neuro-otology Unit, Department of Neuro-surgery, Sanjay Gandhi Post Graduate Institute of Medical Sciences, Raibareily Road, Lucknow (Uttar Pradesh) – 226 014, India; 2 Department of Anesthesiology, Sanjay Gandhi Post Graduate Institute of Medical Sciences, Raibareily Road, Lucknow (Uttar Pradesh) – 226 014, India; 3 Department of Cardiovascular & Thoracic Surgery, Sanjay Gandhi Post Graduate Institute of Medical Sciences, Raibareily Road, Lucknow (Uttar Pradesh) – 226 014, India

## Abstract

**Background:**

Tracheal malignancies are usual victim of delay in diagnosis by virtue of their symptoms resembling asthma. Sometimes delayed diagnosis may lead to almost total airway obstruction. For difficult airways, not leaving any possibility of manipulation into neck region or endoscopic intervention, femorofemoral cardiopulmonary bypass can be a promising approach.

**Case Presentation:**

We are presenting a case of tracheal adenoid cystic carcinoma (cylindroma) occupying about 90% of the tracheal lumen. It was successfully managed by surgical excision of mass by sternotomy and tracheotomy under femorofemoral cardiopulmonary bypass (CPB).

**Conclusion:**

Any patient with recurrent respiratory symptoms should be evaluated by radiological and endoscopic means earlier to avoid delay in diagnosis of such conditions. Femorofemoral cardiopulmonary bypass is a relatively safe way of managing certain airway obstructions.

## Background

Adenoid cystic carcinomas (cylindromas) of the trachea are very rare slow growing tumors and cause symptoms by virtue of their location and size. They are usually treated for many years as asthma unless some enthusiastic surgeon advises CT scan or performs endoscopy and surprisingly finds an intraluminal tracheal mass responsible for the symptoms. They usually become symptomatic when they start obstructing more than 75% of the tracheal lumen.

The lower the mass is present, the more complicated is the anesthesia and the surgery for successful and safe removal of the mass.

Here we are presenting a case of a 31 yrs. old female presenting with difficulty in speaking and breathing and found to have an intratracheal mass situated in thoracic trachea about 3 cms above the carina, occupying about 90% of the lumen. The case was successfully managed with femorofemoral cardiopulmonary bypass, sternotomy, tracheotomy and excision of mass.

## Case presentation

### Case report

A 31 yrs. old female was referred to us with the complaints of difficulty in speaking and progressively increasing difficulty in breathing since 3 yrs. The patient was being treated as asthmatic at some other hospital (with i.v. steroids, antibiotics, nebulizations and bed rest) since the onset of symptoms which relieved the symptoms but recurrence occurred after stopping the treatment.

**Figure 1:**
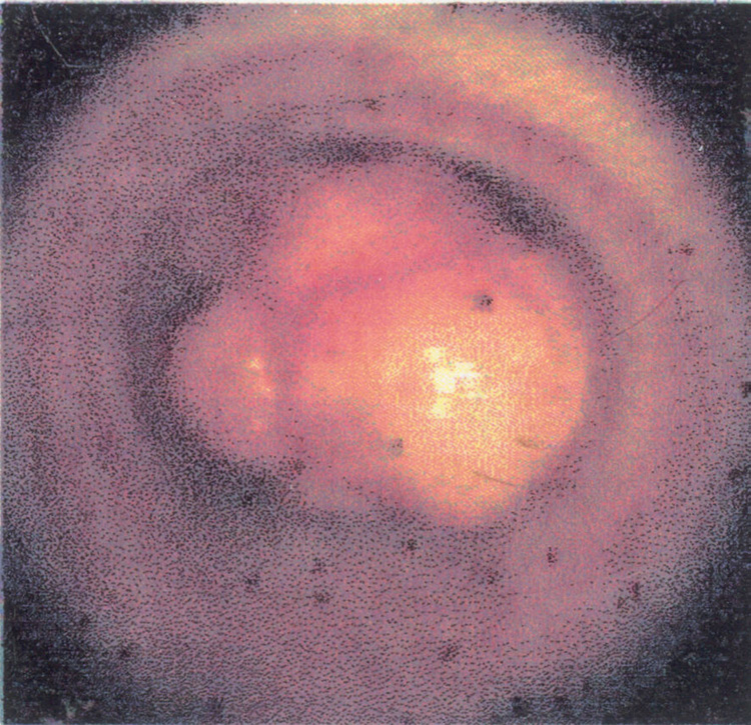
Fibreoptic endoscopic image showing a pedunculated mass causing significant lumen obstruction.

On examination, patient had a weak voice and was having stridor of grade II which increased with extension of neck or in fully supine position. She had mild tachycardia and tachypnoea. Indirect laryngoscopic examination was normal. She was maintaining adequate oxygen saturation on room air with conservative treatment. Fibreoptic tracheoscopy showed an intraluminal pedunculated tracheal mass which was occupying about 90% of the lumen (Figure 1). Biopsy from the mass was inconclusive.

**Figure 2:**
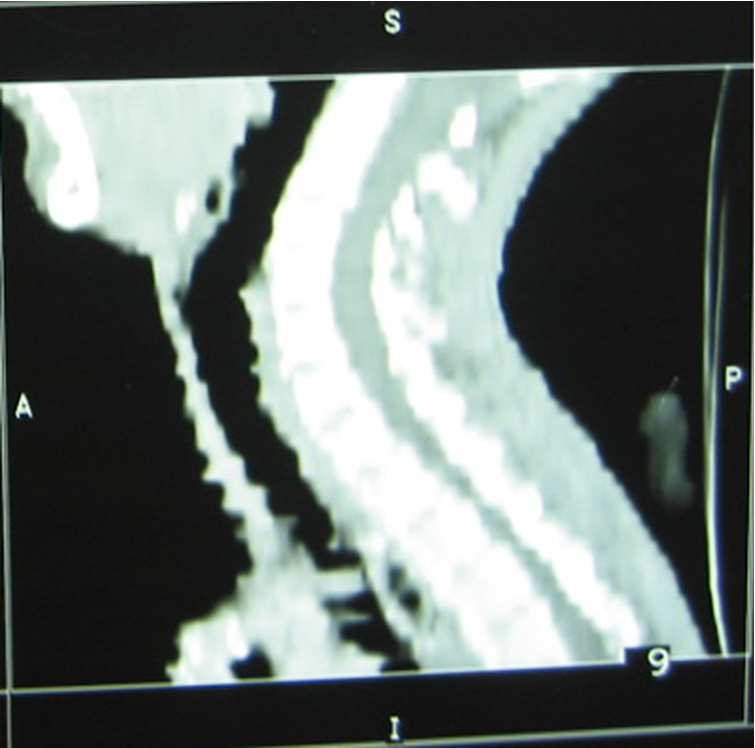
Reconstructed sagittal CT image showing mass into the thoracic trachea.

CT scan of neck and chest revealed a homogenous enhancing pedunculated intraluminal mass, about 2.4 × 1.5 × 1.1 cms in size attached to left lateral wall of the trachea. The peduncle was situated about 3 cms above the carina. About 90% of tracheal lumen was found to be occupied by the mass (Figure 2).

Patient was taken for surgery and a femorofemoral cardiopulmonary bypass was established first for oxygenation of blood. Right femoral artery and vein were accessed and cannulated under local anesthesia and were connected to the cardiopulmonary bypass machine. General anesthesia was then induced and median sternotomy was done. Trachea was exposed up to the carina. There was no extratracheal extension of the mass. The trachea was opened in midline by about 3 cms vertical incision, starting at 2 cms above the carina and the mass was exposed. It was a soft pinkish pedunculated mass attached to left lateral wall of the thoracic trachea.

Mass was removed along with its attachment and adjoining tracheal wall. The tracheal wall surrounding the attachment was normal looking. The resultant small defect in the tracheal wall was repaired with 000 vicryl and sealed with N-butyl cyanoacrylate. The patient was intubated with a cuffed endotracheal tube passing beyond the tracheal incision. Then the patient was taken on ventilator and inhalational anesthetics after weaning off the bypass.

Tracheal incision was sutured & sealed with a thin layer of N-butyl cyanoacrylate. Sternotomy was closed in layers. Right inguinal incision was closed in layers after decannulating the femoral artery and vein and repairing the defects in the vessel walls.

Patient was kept on ventilator in ICU and extubated after 24 hrs.

The histopathology report of the excised mass revealed it to be adenoid cystic carcinoma also known as cylindroma. The patient was referred for radiotherapy.

**Figure 3:**
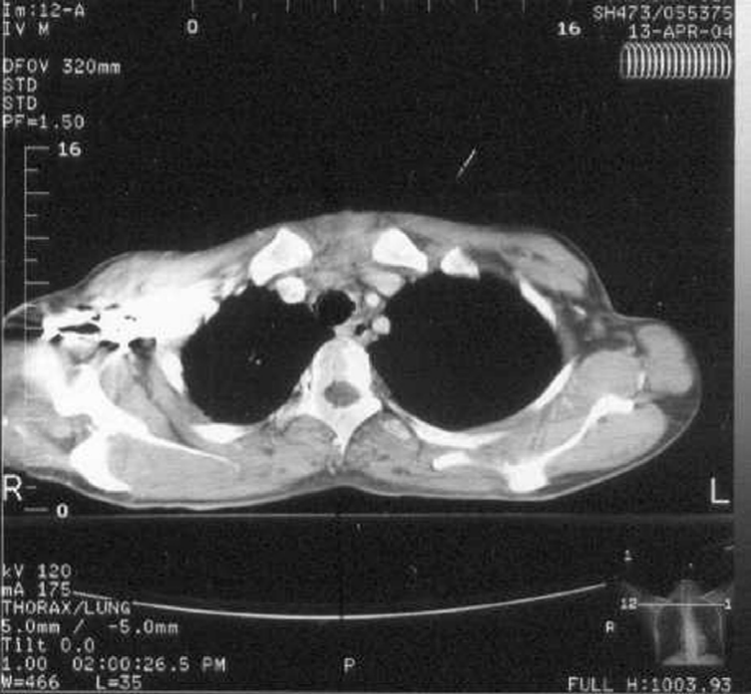
One month postoperative CT chest (axial view) showing normal tracheal walls and lumen.

Repeat CT scan after one month (Figure 3) showed normal tracheal walls and lumen.

## Discussion

Primary tracheal masses are very rare and mostly malignant, occurring in 0.2 per 1, 00,000 persons per year [1] and among these squamous cell carcinomas form the main bulk. Next common primary tracheal malignancy is adenoid cystic carcinoma (cylindroma). Adenoid cystic carcinoma is a slow growing tumor with relatively good prognosis. It mainly involves the salivary glands. Its incidence in the lower airways is low (0.1% of all broncho-pulmonary neoplasms) [2].

The anesthesia in these cases is very challenging as the compromised airway is to be shared by both surgeon and anesthetist. For such cases, cardiopulmonary bypass standby after femoral artery and vein cannulation and then intravenous/inhalational induction while oxygenating the patient with the oxygen inhalation has been considered to be a reasonable approach [3]. In >90% tracheal obstruction cases, IV induction is the preferred method after patient been taken on pump by which anesthesia can be maintained.

We decided to go directly for femorofemoral cardiopulmonary bypass for induction and oxygenation as patient was not tolerating extension of the neck due to degree of tracheal obstruction, so even slight intraoperative neck or tracheal manipulation was anticipated to be hazardous. Byrne JG et al (2004) advocated planned use of CPB to facilitate complete resection of thoracic malignancies after careful patient selection [4]. The availability of CPB also provides a safety net in the event of injury to vascular structures during tumor resection. It has been advocated as safe and preferred method in difficult airway or near total airway obstruction situations [5-8].

Despite chances of excessive bleeding during surgery due to use of anticoagulants in CPB, it seems to be the safest way to provide anesthesia in such cases. Bleeding was not troublesome in our case as we used only partial heparinization (1 mg/kg body weight) instead of full heparinization (3 mg/kg body weight). Further to enhance the safety, heparin coated cannulae can also be used. The femoral vein cannula goes up to the junction of inferior vena cava and right atrium, while femoral artery cannula goes up to abdominal aorta.

Care was taken to maintain pump compatibility with the beating heart to avoid fall in perfusion, especially in the peripheral organs. So bypass was used only as a supportive mean for oxygenation of the blood. Circulation of the blood was maintained by the beating heart and complete emptying of the heart was avoided carefully, as done in ECMO (Extra Corporeal Membrane Oxygenation).

Tracheostomy or endotracheal intubation was not possible due to site and size of lesion and the amount of airway obstruction caused by it. In using high frequency jet ventilation with a small bore catheter, there was a risk of distal dislodgement of any broken piece of mass or increase in airway obstruction by movement of the mass itself, due to pedunculated nature of the mass, and we had nothing safe in our hands to manage that situation. Further in jet ventilation, there was a fair chance of compromising the oxygenation by collapse of the tracheal lumen distal to the mass during expiration or by development of pneumothorax.

Endoscopic and laser excision were not opted due to problems of airway control and anesthesia during the procedure which may provide life threatening acute airway obstruction due to bleeding, secretions, edema or from the bronchoscope itself. These complications were more probable in a pedunculated mass due to possible movement of the mass during manipulation. Furthermore endoscopic laser excision is a better choice for palliation in advanced malignancies [3].

Resection and reconstruction followed by adjuvant radiotherapy provides best chance of cure in tracheal cylindromas [1,8]. For a benign looking, pedunculated, slow growing mass without any obvious wall infiltration or surrounding structure involvement, it was decided to excise only the tumor attachment instead of going for complete resection of a segment of trachea and anastomosis. Donna E. Maziak et al (1996) found that there was only a small difference in survival between patients having complete and incomplete resection for tracheal cylindroma [8].

## Conclusion

It can be concluded that it is always prudent to go for endoscopic or radiological evaluation of airways in patients with long standing or recurrent respiratory symptoms. Also even benign looking tracheal masses can come out to be malignant and should be sought with suspicion. Furthermore femorofemoral cardiopulmonary bypass is an effective, safe and convenient method for anesthesia for intraluminal tracheal masses in thoracic trachea with significant obstruction, allowing the surgeon enough freedom and comfort during surgery.

## Competing interests

The author(s) declare that they have no competing interests.

## Authors' contributions

All five authors

1) Have made substantial contributions in management of this case and in conception, design, analysis and interpretation of results of this case report

2) Have been involved in drafting the article or revising it critically for important intellectual content.

3) Have given final approval to the version to be published

## Pre-publication history

The pre-publication history for this paper can be accessed here:
